# Blood co-expression modules identify potential modifier genes of diabetes and lung function in cystic fibrosis

**DOI:** 10.1371/journal.pone.0231285

**Published:** 2020-04-17

**Authors:** Fanny Pineau, Davide Caimmi, Milena Magalhães, Enora Fremy, Abdillah Mohamed, Laurent Mely, Sylvie Leroy, Marlène Murris, Mireille Claustres, Raphael Chiron, Albertina De Sario

**Affiliations:** 1 EA7402, Laboratoire de Génétique de Maladies Rares (LGMR), University of Montpellier, Montpellier, France; 2 CRCM, Arnaud de Villeneuve Hospital, Montpellier, France; 3 CRCM, Renée Sabran Hospital, Hyères, France; 4 CRCM, Pasteur Hospital, Nice, France; 5 CRCM, Larrey Hospital, Toulouse, France; 6 CHU Montpellier, Laboratoire de Génétique Moléculaire, Montpellier, France; Centre National de la Recherche Scientifique, FRANCE

## Abstract

Cystic fibrosis (CF) is a rare genetic disease that affects the respiratory and digestive systems. Lung disease is variable among CF patients and associated with the development of comorbidities and chronic infections. The rate of lung function deterioration depends not only on the type of mutations in *CFTR*, the disease-causing gene, but also on modifier genes. In the present study, we aimed to identify genes and pathways that (i) contribute to the pathogenesis of cystic fibrosis and (ii) modulate the associated comorbidities. We profiled blood samples in CF patients and healthy controls and analyzed RNA-seq data with Weighted Gene Correlation Network Analysis (WGCNA). Interestingly, lung function, body mass index, the presence of diabetes, and chronic *P*. *aeruginosa* infections correlated with four modules of co-expressed genes. Detailed inspection of networks and hub genes pointed to cell adhesion, leukocyte trafficking and production of reactive oxygen species as central mechanisms in lung function decline and cystic fibrosis-related diabetes. Of note, we showed that blood is an informative surrogate tissue to study the contribution of inflammation to lung disease and diabetes in CF patients. Finally, we provided evidence that WGCNA is useful to analyze–omic datasets in rare genetic diseases as patient cohorts are inevitably small.

## Introduction

Cystic fibrosis (CF; OMIM 219700) is an autosomal recessive inherited disease that affects approximately 1/3000 newborns [1]. It results from impairment of the Cystic Fibrosis Transmembrane Conductance Regulator (CFTR) protein, a chloride channel expressed at the apical membrane of various epithelial cells. The defective protein results in thick, sticky and obstructive mucus in multiple organs of the respiratory, digestive and reproductive systems [1,2]. The mutant CFTR protein is also responsible for an altered innate and adaptive immune function.

Lung disease is the main cause of morbidity and mortality in cystic fibrosis [2]. CF patients present chronic infections and abnormal inflammation of the lungs that lead to progressive airway destruction. The rate of lung function deterioration is variable among patients and associated with the development of comorbidities and chronic infections [1,2].

CF-related diabetes (CFRD) is a common comorbidity of CF [3]. It affects about 20% of adolescents and 40% to 50% of adults, and is associated with more frequent pulmonary exacerbations, accelerated pulmonary function decline and higher mortality. CFRD is characterized by a reduced and delayed insulin response. The beta-cell dysfunction is evident before the onset of diabetes and is already associated with a pulmonary function decline [3]. The exact causes of CFRD are not totally elucidated, nor is explained the association between diabetes and accelerated lung function loss. Mutant cftr-/- newborn zebrafishes have fewer beta cells than the wildtype ones, which suggests that the CFTR protein is important for pancreas development [4]. In addition, CFTR seems to be critical for insulin exocytosis, which implies that CF patients have an intrinsic pancreatic islet dysfunction [5]. Finally, the continuous infiltration of immune cells into the pancreas may contribute to the progressive destruction of the islets [6].

The defective CFTR protein and subsequent insufficient mucociliary clearance predispose CF patients to acute and, ultimately, chronic lung infections with opportunistic pathogens [7]. Chronic *P*. *aeruginosa* infection is found in approximately 40% of adult CF patients and is also associated with a drastic decrease of lung function [7].

Previous genetic studies showed that the clinical variability of CF patients depends not only on the type of mutations in the *CFTR* gene, but also on modifier genes, other genes that modulate the patient phenotype [1]. Much current research focuses on finding CF modifier genes to develop new therapies [1].

In the present study, we analyzed the transcriptome to identify genes and pathways that (i) contribute to the pathogenesis of cystic fibrosis and (ii) modulate the associated comorbidities. Knockout mice models have limitations because they do not develop spontaneous diabetes, nor do they present lung disease [8]; on the other hand, human pancreas and airway tissues are not easily accessible for studies. Herein, we used whole blood samples from CF patients as a surrogate tissue. Of interest, we found that lung function, body mass index (BMI), the presence of diabetes, and chronic *P*. *aeruginosa* infection correlated with modules of co-expressed genes.

## Materials and methods

### Study population

The study population included ≥ 18-year old subjects from the MethylCF cohort: 33 cystic fibrosis patients and 16 healthy controls [9,10]. A replication set of subjects from the same cohort was used for real-time PCR validation (20 CF patients and 8 healthy controls). The two sets were similar with respect to the age and male-to-female ratio. Demographic and clinical features are reported (**Table 1**). CF patients carried the homozygous p.Phe508del mutation. The presence of diabetes was determined on the basis of an abnormal oral glucose-tolerance test. CF patients were classified as chronically infected by P. *aeruginosa*, methicillin-resistant S. *aureus* and/or A. *fumigatus*, whenever they had three consecutive positive sputum cultures after antibiotic treatment. The study was approved by the “Comité de Protection des Personnes Sud Méditerranée III” Institutional Review Board (2013.02.01bis) and is registered at clinical.gov under reference #NCT02884. Informed written consent was obtained from all participants.

**Table 1:** Demographic and relevant clinical features of the MethylCF cohort.

	Discovery set		Replication set	
	Controls	CF patients	Controls	CF patients
	(n = 16)	(n = 33)	(n = 8)	(n = 20)
Age, years†	28	28 (10)	31	25 (8)
Sex, M:F	10:6	23:10	3:5	10:10
BMI, kg/m^2^†		21 (4)		21 (4)
Weight, kg†		60 (13)		60 (12)
Height, cm†		171 (9)		168 (11)
FEV_1_, %†		48 (24)		48 (24)
FEV_1_, L†		1.91 (1.1)		1.70 (0.8)
FVC, %†		76 (22)		68 (28)
FVC, L†		3.32 (1.1)		2.99 (0.6)
PI, %		100		100
Diabetes, %*		36		30
HbA1c, %†		6.1 (1.1)		5.4 (na)
Atopy, %		18		45
*P*. *aeruginosa*, %§		94		95
*MRSA*, %§		36		15
*A*. *fumigatus*, %		21		25
Azythromycin, %		91		100
Aztreonam, %		12		25
Colistin, %		39		50
Tobramycin, %		55		50
Corticosteroid, %		33		45

BMI, body mass index; CF, cystic fibrosis; FEV_1_, forced expiratory volume in 1 second

FVC, forced vital capacity; HbA1c, glycated hemoglobin fraction; PI, pancreatic insufficiency

MRSA, Methicillin-resistant *Staphyloccocus aureus*.

† Median values (interquartile range).

* The presence of diabetes was determined on the basis of an abnormal oral glucose-tolerance test.

§ CF patients were classified as chronically infected by P. *aeruginosa*, MSRA and/or A. *fumigatus*

whenever they had three consecutive positive sputum cultures after antibiotic treatment.

na, not applicable because only five measurements were available.

### RNA sequencing and differential expression analysis

RNA was extracted from whole blood samples using the PAXgene Blood RNA kit (#762124, PreAnalytix), according to the manufacturer’s recommendations [9]. Total RNA sequencing libraries were prepared with the TruSeq Stranded Total RNA kit (Illumina®) and ribosomal RNA was depleted using the Ribo-Zero Gold rRNA removal kit following the manufacturer's instructions. Libraries were sequenced in paired-end 75 nucleotides mode with a HiSeq4000 Illumina. The quality of raw sequenced reads was assessed using the FASTQC quality control tool and reads were mapped to the reference human genome build hg19/GRCh37 with Tophat 2 [11]. We used HTSeq to obtain the number of reads associated with each gene in the Gencode v26lift37 database (restricted to protein-coding genes, antisense and lincRNAs) [12]. The differential expression of the annotated genes was calculated using DESeq [13]. Transcripts with a minimum 2-fold change and a Benjamin-Hochberg adjusted p-value (FDR) < 0.05 were considered as differentially expressed. Normalized data and raw data generated during the current study are available in Gene Expression Omnibus (GEO) with accession number 136371 (https://www.ncbi.nlm.nih.gov/geo/query/acc.cgi?acc=GSE136371).

### Real-time PCR validation

Total RNA from whole blood samples were reverse transcribed from 500 ng as previously described [9]. Primers were designed using Primer3Plus and Beacon Designer Free Edition online tools, using a Gibbs energy threshold of ΔG ≥ -2.0 for hetero- and homodimers and a GC content > 40% (**S1 Table**). All primers were tested to display an efficiency of amplification of at least 93% (±SD 6%). Amplicons overlapped exon junctions except for CITF22-49E9.3. Real-time PCR reactions were done in duplicate in two independent reverse transcriptions, using SYBR Green I Master mix (Roche Diagnostics) and a LightCycler 480 Instrument. The reverse transcription reaction program consisted of 10 min pre-incubation at 95°C followed by a three step amplification (95°C for 10 s, 60°C for 20 s, 72°C for 10 s). Standard curves were generated by serial dilutions of a control cDNA. Expression levels were expressed as ratios relative to that of the reference gene (*YWHAZ*), using the Pffafl method (with efficiency correction) [14]. Differences between groups were analyzed with Wilcoxon test and were considered significant when p-value < 0.05.

### Weighted Gene Correlation Network Analysis

To identify modules of co-expressed genes, we implemented Weighted Gene Correlation Network Analysis in the WGCNA R package [15]. We used the WGCNA functions to (i) construct a network of coexpressed and highly connected genes, (ii) identify modules of coexpressed genes, and (iii) correlate the gene modules with biological features (continuous or binary phenotypic traits) of the MethylCF cohort. Unless otherwise specified below, we used the default parameters as described by [15].

Briefly, we preselected a list of 15077 genes with a FPKM > 0.1 and log2-transformed the FPKM values (FPKM value +1). Then, we generated a signed adjacency matrix using the biweight midcorrelation and raising it to the power beta = 18 to reduce the noise. The adjacency network exhibited approximate scale-free topology (R^2^ = 0.92). Scale-free topology is obtained when few genes are highly connected to each other (hub genes), whereas the remaining genes are weakly connected. The adjacency matrix was transformed into a topological overlap matrix, an adjacency matrix that considers coexpression information and topological similarity. Modules were generated using the dynamic tree cut and modules with highly correlated module eigengenes (correlation > 0.75) were merged together. Correlations between the modules of co-expressed genes (eigengenes) and clinical and demographic features of the MethylCF cohort were calculated. Top genes in the modules were visualized with Cytoscape [16].

### Gene ontology analysis

Gene ontology (GO) and KEGG pathways were analyzed with WebGestalt (WEB-based GEne SeT AnaLysis Toolkit; URL: http://www.webgestalt.org) using the Benjamini–Hochberg correction for multiple testing [17]. For GO, we retained a false discovery rate of 5%, excluding categories with less than four genes. For KEGG pathway analyses, we used a false discovery rate of either 1% or 5%.

## Results

### RNA sequencing and biotype distribution

Whole blood samples had been collected from CF patients with no ongoing pulmonary exacerbation [9]. Total blood cell count and the percentage of different types of leukocytes were within normal range. The median percentages calculated on 19 CF patients were 60% (iqr 14.7%) neutrophils, 26% (iqr 12.0%) lymphocytes, 9% (iqr 3.0%) monocytes, 3% (iqr 2.6%) eosinophils, 1% (iqr 0.5%) basophils. We collected the blood samples in PAXgene tubes that stabilize RNA, preserve all types of circulating cells (leukocytes and platelets) and do not alter the gene expression profile of frozen samples [18].

Using RNA-seq, we generated the transcriptome of 49 blood samples from the MethylCF cohort [9,10]. Eight samples were excluded because ribosomal depletion failed. Hence, the bioinformatic analyses were carried out on 41 samples (27 CF patients and 14 controls). The median total number of reads per sample was 112 million (iqr 17 million). We found that 9324 protein coding genes, 501 antisense transcripts and 493 lincRNA were expressed in blood samples (FPKM >1). Biotype distribution and expression level of the corresponding transcripts are represented in **S1 Fig**.

### Differentially expressed genes between CF patients and controls

**Fig 1:**
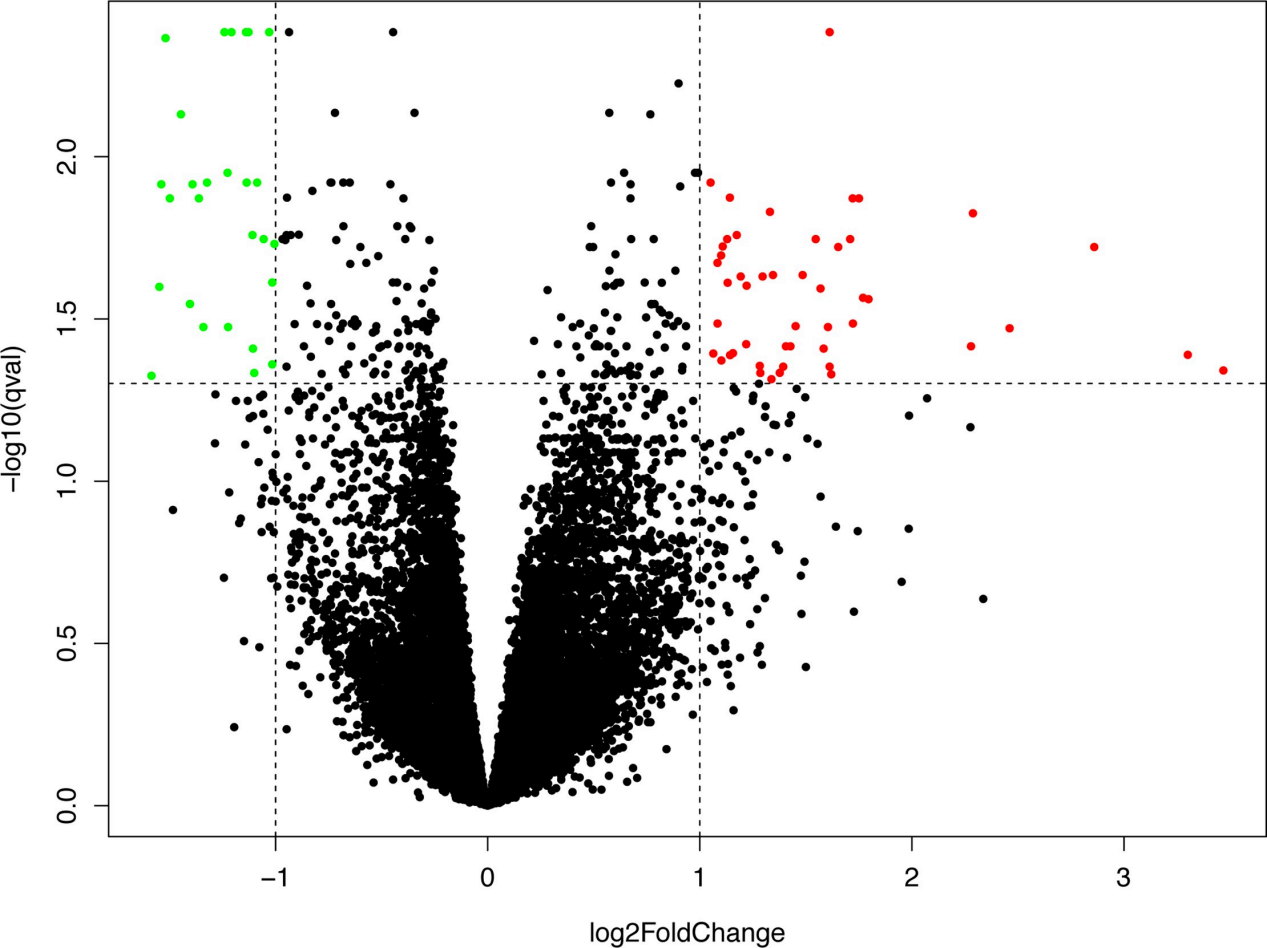
Differentially expressed gene between CF patients and controls. The volcano plot represents the Log_2_ transformed fold-changes (x-axis) and the Log_10_ transformed q-values (y-axis). 48 genes were over-expressed (red) and 27 genes were under-expressed (green) in CF blood samples (FDR < 0.05).

When we compared gene expression between CF patients and controls, we found 75 differentially expressed (DE) genes (48 genes were over-expressed and 27 genes were under-expressed in CF patients) (Log_2_foldChange ≥ 1 or ≤ -1; FDR < 0.05) (**Fig 1**, **S2 Table**). Thirteen non-coding RNAs were over-expressed and two non-coding RNAs were under-expressed in CF patients compared to controls (**S2 Table**).

Gene ontology (GO) analysis showed that DE genes between CF patients and controls were overrepresented among genes important for the response to bacterial infection (FDR p-value = 1.2E-05, 19 genes including *TLR5*, *S100A8*, *S100A12*, *ILR23R)* and leukocyte activation (FDR p-value = 3.4E-04, 11 genes including *IL23R*, *IL4R*, *CDC80*, *TBX21*) (**S3 Table)**. KEGG analysis highlighted the Th17 lymphocyte activation pathway (FDR p-value = 4.6E-03, 7 genes: *MAPK14*, *IL23R*, *TBX21*, *HLA-DOA*, *IL2RB*, *IL4R*, *RORC)*.

### Validation of RNA-seq data with real-time PCR

To validate the RNA-seq data, we assessed the expression levels of three DE genes (*TLR5*, *CLEC4D*, and *ALPL*) and one DE lincRNA (CITF22-49E9.3) using real-time PCR in the same set of blood samples (n = 49) as a technical validation, and in a replication set (n = 28) as a biological validation (**Table 2**). We selected protein-coding and non-coding transcripts among the top DE genes with a relevant biological function and a range of expression levels (from 5 to 56 FPKM). *TLR5*, *CLEC4D*, *ALPL* and CITF22-49E9.3 were differentially expressed between CF patients and controls of the discovery set, and thus technically validated (p-value < 0.01). *TLR5*, *CLEC4D*, and *ALPL* were biologically validated since their expression levels differed between CF patients and controls of the replicative set (p-value < 0.05). CITF22-49E9.3 failed to be biologically validated (p-value = 0.18), but the direction was the same as in RNA-seq (over-expression in CF). For all loci, the direction of differential expression was identical and fold-changes were similar between RNA-seq and real-time PCR data, showing a total concordance between the two techniques (**Table 2**).

**Table 2:** Validation of RNA-seq data by real time-PCR.

	*RNA-seq—*Discovery set				*real time-PCR*—Discovery set				*real time-PCR—*Replication set			
	FPKM		FC	p-value†	Normalized ratios		FC	p-value†	Normalized ratios		FC	p-value†
	Med CF	Med C	Med CF	Med C	Med CF	Med C
**TLR5**	13.87	4.69	2.96	3.9E-05	2.59	1.15	2.26	6.6E-05	2.72	1.39	1.96	3.9E-03
**CLEC4D**	14.70	8.73	1.68	3.5E-05	5.72	2.60	2.20	2.6E-06	4.68	2.61	1.79	1.6E-02
**ALPL**	49.21	19.32	2.55	7.9E-04	2.34	0.84	2.80	7.3E-03	2.88	0.91	3.18	2.1E-03
**CITF22-49E9.3**	9.35	5.38	1.74	6.1E-05	3.37	2.09	1.61	1.5E-06	3.15	2.20	1.43	1.8E-01

FPKM: Fragments Per Kilobase of transcript per Million mapped reads, Med: median, CF: Cystic Fibrosis, C: Control, FC: fold-change (CF/C).

† CF vs C, Wilcoxon test.

### Weighted Gene Correlation Network Analysis

Next, to find additional genes important for CF pathogenesis and the associated comorbidities, we implemented Weighted Gene Correlation Network Analysis (WGCNA) [15]. Using RNA-seq datasets from 27 CF patients of the MethylCF cohort, we found 28 modules of co-expressed genes (**S4 Table**). The number of genes in each module ranged from 35 to 2839. A majority of modules were enriched with genes that belong to biological pathways (Notch signaling, MAPK signaling, platelet activation, B cell receptor, etc), which suggests that the gene modules are biologically meaningful (**Table 3**).

**Table 3:** Module of coexpressed genes: KEGG analysis.

	Size*	KEGG §	Pathway	N	R	FDR
Darkgreen	90					n.s.
Red	802	hsa04330	Notch signaling pathway	48	6.9	4.3E-04
Turquoise	2838	hsa04666	Fc gamma R-mediated phagocytosis	91	6.7	8.0E-10
Black	679	hsa00310	Lysine degradation	59	6.5	4.2E-03
Tan	243	hsa05210	Colorectal cancer	86	7.8	1.8E-02
White	35	hsa04120	Ubiquitin mediated proteolysis	137	10.2	6.8E-03
Orange	66	hsa04217	Necroptosis	162	12.1	1.3E-06
Royalblue	140	hsa04621	NOD-like receptor signaling	168	8.6	1.8E-06
Darkred	107					n.s.
Greenyellow	320	hsa04932	Non-alcoholic fatty liver disease	149	7.1	2.5E-06
Purple	573	hsa04144	Endocytosis	244	3.3	3.2E-04
LightYellow	143	hsa04611	Platelet activation	122	8.2	9.3E-05
Magenta	595					n.s.
Salmon	235					n.s.
Cyan	173	hsa03050	Proteasome	44	20.4	1.0E-05
Darkgrey	68	hsa05203	Viral carcinogenesis	201	14.2	0.0E+00
Lightgreen	145					n.s.
Pink	673	hsa04010	MAPK signaling	255	2.8	1.0E-03
Darkturquoise	79					n.s.
Grey60	156	hsa01230	Biosynthesis of amino acids	75	8.2	6.7E-03
Darkorange	59					n.s.
Midnightblue	171	hsa04650	Natural killer cell mediated cytotoxicity	131	6.3	0.0E+00
Brown	1484	hsa03010	Ribosome	154	12.2	0.0E+00
Green	1097	hsa03010	Ribosome	153	6.4	1.6E-12
Lightcyan	169	hsa04662	B cell receptor signaling	71	16.2	7.8E-06
Blue	1859	hsa04660	T cell receptor signaling	101	4.9	2.6E-02
Yellow	1320					n.s.
Grey	757					n.s.

*Number of genes in the module

§ Top KEGG pathway

N, number of genes in the pathway. R, ratio of enrichment. FDR, false discovery rate

n.s., not significant, p-value > 0.05 after Benjamini-Hochberg correction

**Fig 2:**
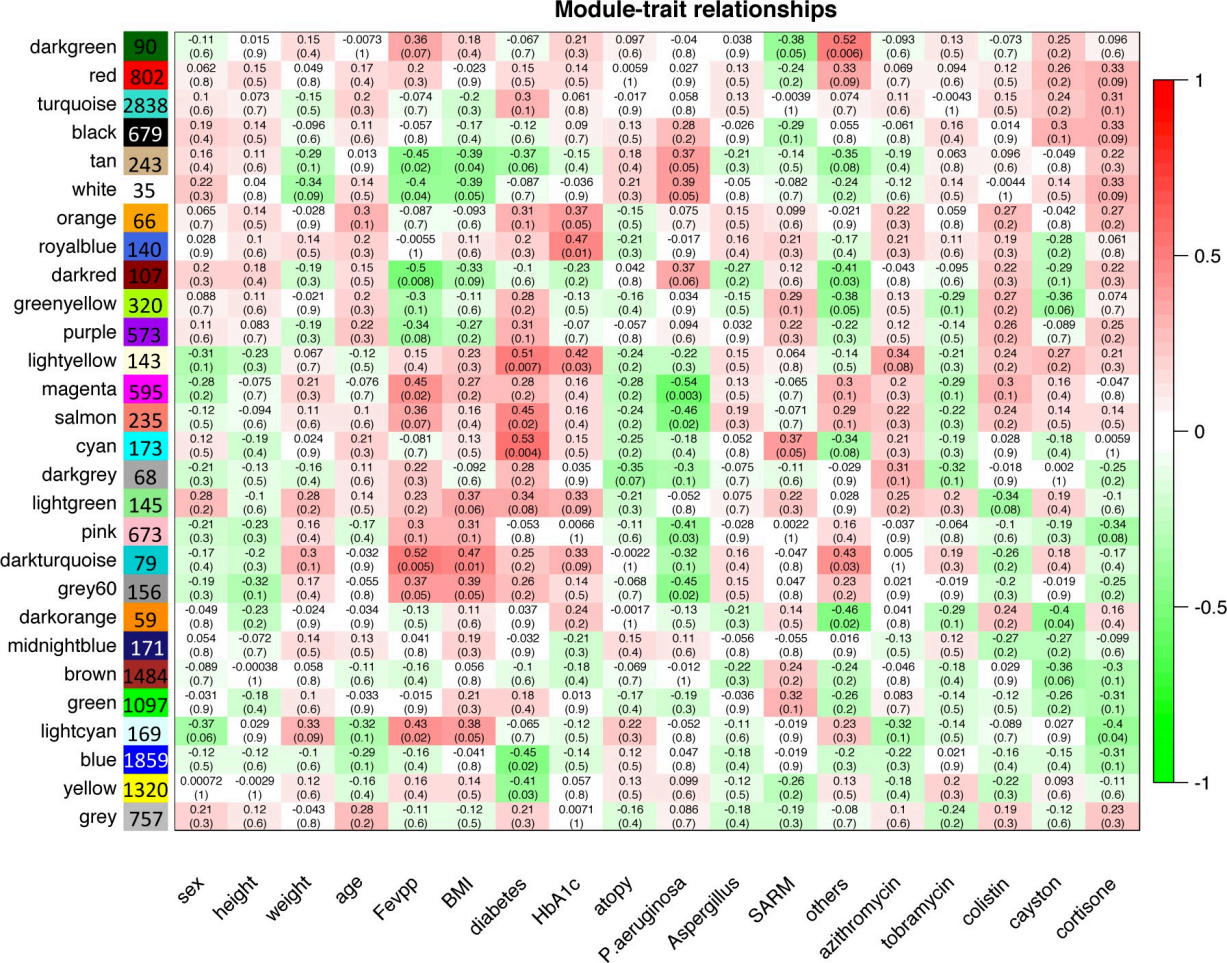
WGCNA on blood RNA-seq dataset. The heatmap represents the correlation (coefficient and p-value) between eigengene modules and clinical traits. Module sizes are shown in the colored boxes.

Next, we calculated the correlation between the clinical traits of the patients and the eigengenes of the modules (**Fig 2)**. The eigengene is the first component of a principal component analysis and represents the summary of the gene expression profile of the module [15]. Modules of co-expressed genes that correlated with lung function, the presence of diabetes, and a chronic *P*. *aeruginosa* infection were analyzed in more detail.

**Fig 3:**
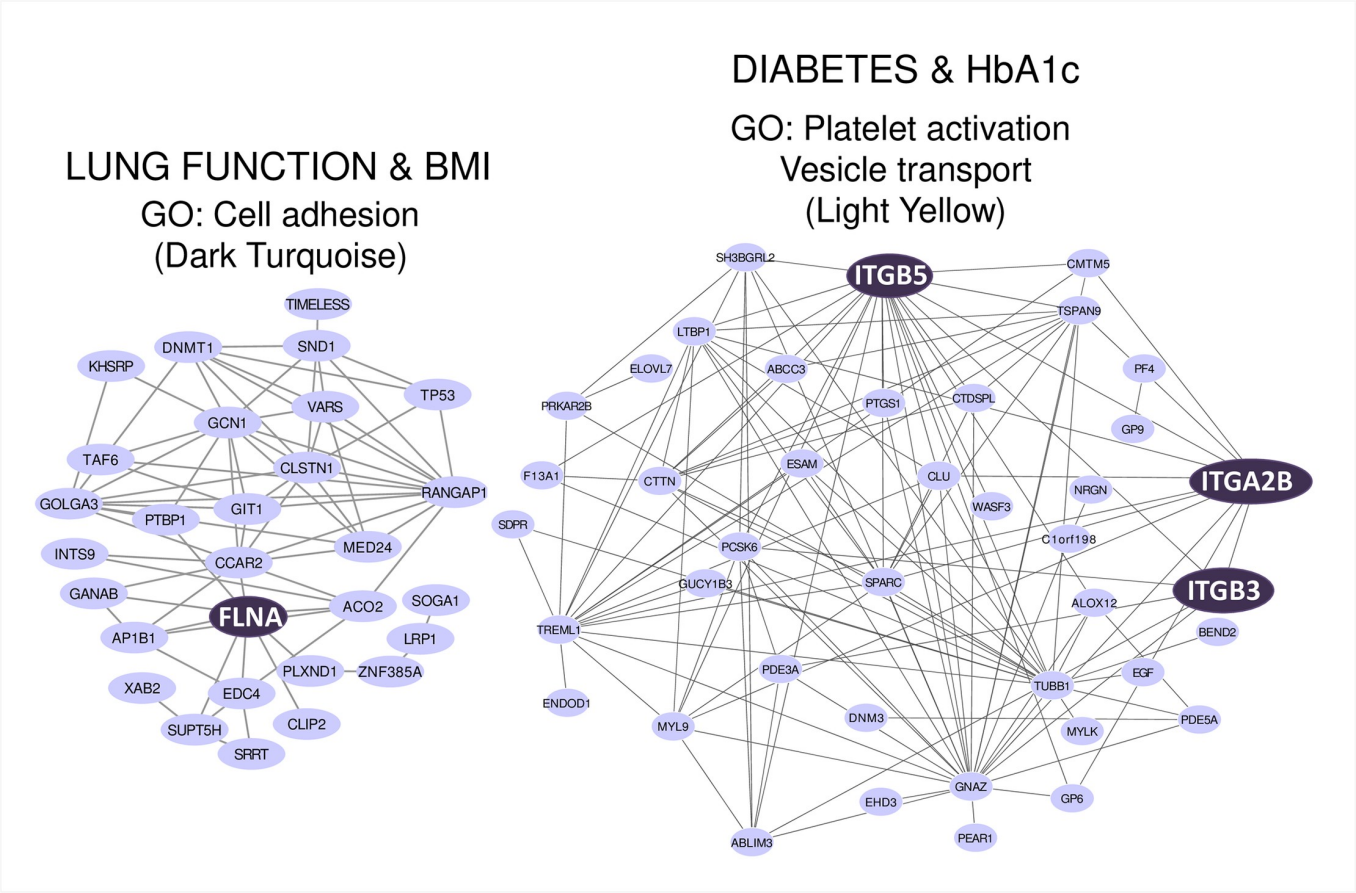
Network representation of dark turquoise and light yellow modules. Top genes and their connections were visualized with Cytoscape [16]. Genes of interest were emphasized. For each module the correlated clinical traits and main gene ontology (GO) terms are shown.

Lung function metrics included forced expiratory volume in 1 second (FEV_1_) and forced vital capacity (FVC) expressed in liters and as a percent predicted based on age, sex and height [19]. These clinical measures are used for the follow-up of CF patients and as endpoints to assess whether patients respond to treatments [20]. In the MethylCF cohort, lung function and BMI best correlated with the dark turquoise module. For simplicity, only FEV_1_ (%) was represented (**Fig 2**), however, consistent results were obtained with FVC (%) (r = 0.54 p-value = 4.0E-03). The GO analysis of the dark turquoise module highlighted terms related to cell-cell adhesion and cell adherence junctions (**Table 4**). *FLNA* is a hub gene of this module. It encodes Filamin A, an actin-binding and scaffolding protein that interacts with integrins to regulate leukocyte trafficking (**Fig 3**) [21].

**Table 4:** GO terms for dark turquoise module correlated with lung function and BMI, and light yellow module correlated with diabetes.

Geneset	Description	Number of genes	Ratio of enrichment	FDR
**DARKTURQUOISE MODULE**				
***BIOLOGICAL PROCESS***				n.s.
CELLULAR COMPONENT*				
GO:0030529	intracellular ribonucleoprotein complex	12	4.4	7.7E-03
GO:1990904	ribonucleoprotein complex	12	4.4	7.7E-03
GO:0005912	adherens junction	11	4.3	1.0E-02
GO:0061695	transferase complex, transferring phosphorus-containing groups	7	7.5	1.0E-02
GO:0070161	anchoring junction	11	4.2	1.0E-02
GO:0005730	nucleolus	12	3.8	1.1E-02
GO:1990234	transferase complex	11	4.0	1.2E-02
GO:0044798	nuclear transcription factor complex	5	10.1	1.7E-02
GO:0005913	cell-cell adherens junction	7	5.9	2.0E-02
GO:1902494	catalytic complex	14	3.0	2.0E-02
***MOLECULAR FUNCTION***				
GO:0003723	RNA binding	17	2.8	3.8E-02
GO:0044877	macromolecular complex binding	15	3.0	3.8E-02
GO:0098641	cadherin binding involved in cell-cell adhesion	7	6.5	3.8E-02
GO:0098632	protein binding involved in cell-cell adhesion	7	6.2	3.8E-02
GO:0044822	poly(A) RNA binding	14	3.1	3.8E-02
GO:0098631	protein binding involved in cell adhesion	7	6.1	3.8E-02
GO:0045296	cadherin binding	7	6.1	3.8E-02
***KEGG PATHWAY***				n.s.
**LIGHT YELLOW MODULE**				
BIOLOGICAL PROCESS*				
GO:0007596	blood coagulation	34	4.2	6.8E-09
GO:0042060	wound healing	43	3.4	6.8E-09
GO:0050817	coagulation	34	4.1	6.8E-09
GO:0007599	hemostasis	34	4.1	6.8E-09
GO:0009611	response to wounding	44	2.9	3.4E-07
GO:0030168	platelet activation	20	5.1	3.5E-06
GO:0050878	regulation of body fluid levels	35	2.9	1.4E-05
GO:0002576	platelet degranulation	15	6.0	3.2E-05
GO:0070527	platelet aggregation	10	7.2	9.3E-04
GO:0006887	exocytosis	27	2.8	1.4E-03
CELLULAR COMPONENT*				
GO:0031091	platelet alpha granule	16	11.4	4.9E-10
GO:0031410	cytoplasmic vesicle	73	2.3	6.1E-09
GO:0097708	intracellular vesicle	73	2.3	6.1E-09
GO:0044433	cytoplasmic vesicle part	49	2.8	8.4E-09
GO:0031093	platelet alpha granule lumen	12	11.7	6.4E-08
GO:0099503	secretory vesicle	28	3.3	8.0E-06
GO:0034774	secretory granule lumen	12	7.6	8.4E-06
GO:0030141	secretory granule	23	3.5	3.0E-05
GO:0005925	focal adhesion	24	3.3	3.9E-05
GO:0005924	cell-substrate adherens junction	24	3.3	3.9E-05
***MOLECULAR FUNCTION***				n.s.
***KEGG PATHWAY***				
hsa04611	Platelet activation—Homo sapiens (human)	10	8.2	9.3E-05

* Top 10 enriched terms

n.s., not significant after Benjamini-Hochberg correction

Diabetes and glycated hemoglobin (HbA1c) levels correlated with the light yellow module. HbA1c reflects the mean glucose levels over a three-month period. GO analysis of this module revealed terms related to vesicle transport and platelet activation (**Table 4**). Among the hub genes of this network were integrin genes (*ITGB3*, *ITGA2B*, *ITGB5*) (**Fig 3**).

**Fig 4:**
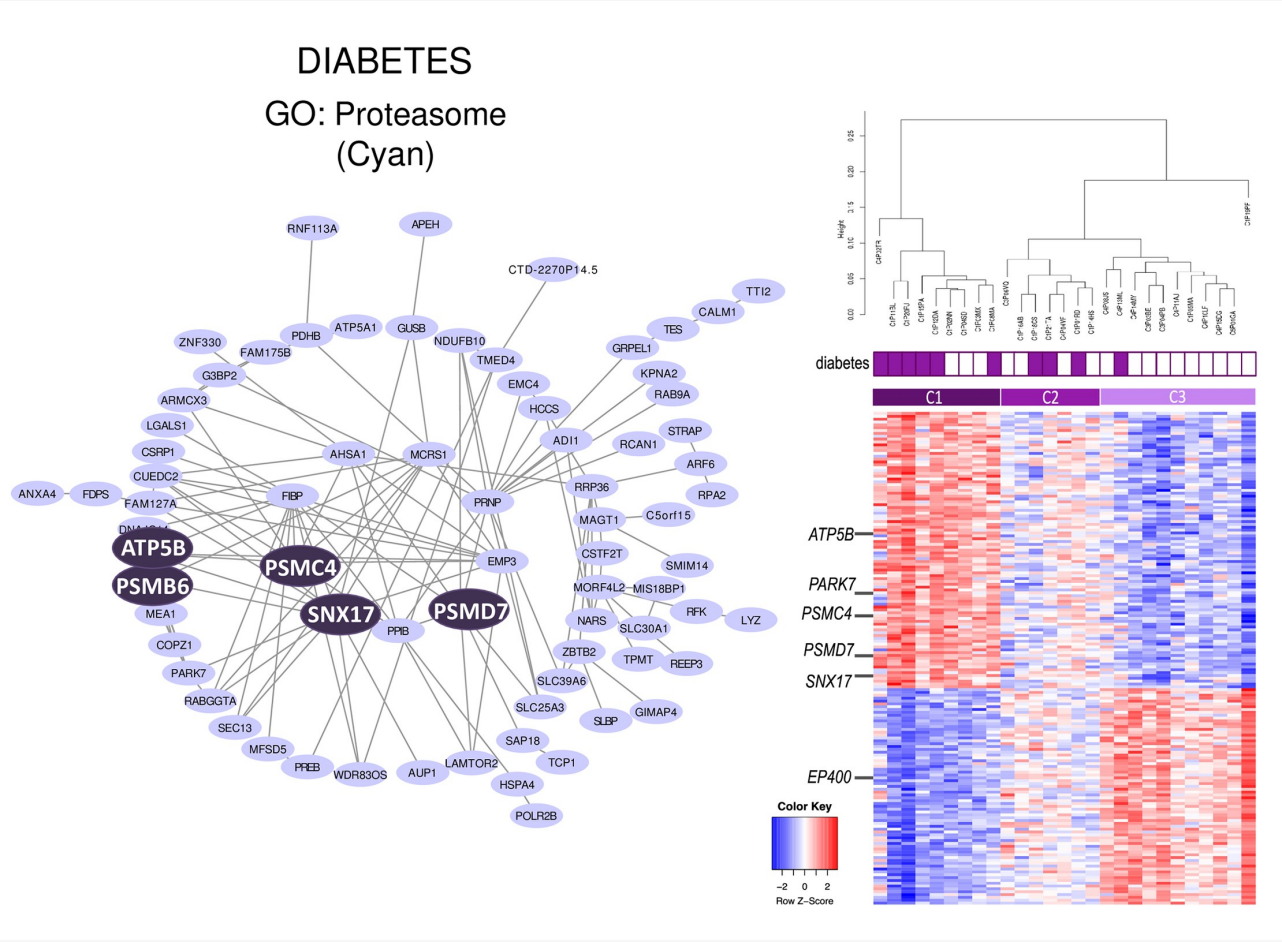
Network representation of cyan module. Top genes and their connections were visualized with Cytoscape [16] (left side). Genes of interest were emphasized. The correlated clinical trait and main gene ontology (GO) term are shown. Hierarchical clustering and heatmap of the cyan genes: purple square, CF patients with diabetes; white square, CF patients without diabetes. C1, C2, C3: cluster 1, 2 and 3, respectively (right side).

The presence of diabetes (but not HbA1c levels) also correlated with the cyan module. GO analysis of the genes belonging to this module revealed enrichment for terms related to the proteasome (**Table 5**). Through hierarchical clustering, we identified three groups of CF patients with distinct gene expression signatures and prevalence of diabetes (**Fig 4**). In the group enriched with diabetic patients (6 out of 9 patients), seven proteasome genes (*PSME4*, *PSMA7*, *PSMB4*, *PSMB6*, *PSMC4*, *PSMD7* and *PSMD8*) and three genes previously associated with common diabetes (*SNX17*, *PARK7/DJ-1* and *ATP5B)* were highly expressed (**Fig 4**). By contrast, two genes encoding histone methyltransferases (*SMYD3* and *KMT2A*) and one gene encoding a chromatin-remodeling ATPase (*EP400)* were under-expressed in this group of patients.

**Table 5:** GO terms for cyan module correlated with diabetes and magenta module correlated with *P*. *aeruginosa* infection.

Geneset	Description	Number of genes	Ratio of enrichment	FDR
**CYAN MODULE**				
***BIOLOGICAL PROCESS***				
GO:0042180	cellular ketone metabolic process	9	8.6	7.0E-04
GO:0009308	amine metabolic process	7	12.0	7.0E-04
GO:0006520	cellular amino acid metabolic process	10	5.8	1.9E-03
GO:0038061	NIK/NF-kappaB signaling	6	11.5	2.6E-03
GO:0007164	establishment of tissue polarity	6	10.6	3.2E-03
GO:0001738	morphogenesis of a polarized epithelium	6	9.6	4.7E-03
GO:0010608	posttranscriptional regulation of gene expression	10	4.7	4.9E-03
CELLULAR COMPONENT*				
GO:0000502	proteasome complex	6	25.5	6.3E-05
GO:1905369	endopeptidase complex	6	25.5	6.3E-05
GO:1902494	catalytic complex	18	4.0	8.0E-05
GO:1905368	peptidase complex	6	18.9	1.9E-04
GO:0005844	polysome	4	25.5	3.7E-03
GO:0030529	intracellular ribonucleoprotein complex	11	4.2	7.0E-03
GO:1990904	ribonucleoprotein complex	11	4.2	7.0E-03
GO:0005839	proteasome core complex	3	40.1	7.0E-03
GO:0005838	proteasome regulatory particle	3	38.3	7.2E-03
GO:0022624	proteasome accessory complex	3	33.7	9.6E-03
***MOLECULAR FUNCTION***				
GO:0003723	RNA binding	22	3.7	2.8E-05
GO:0044822	poly(A) RNA binding	19	4.3	2.8E-05
GO:0004298	threonine-type endopeptidase activity	3	37.6	3.0E-02
GO:0070003	threonine-type peptidase activity	3	37.6	3.0E-02
GO:0035257	nuclear hormone receptor binding	5	10.2	4.7E-02
***KEGG PATHWAY***				
hsa03050	Proteasome—Homo sapiens (human)	6	29.6	1.1E-05
**MAGENTA MODULE**				
BIOLOGICAL PROCESS^*^				
GO:0006778	porphyrin-containing compound metabolic process	9	12.9	1.7E-04
GO:0016567	protein ubiquitination	38	2.6	3.2E-04
GO:0030163	protein catabolic process	38	2.5	5.2E-04
GO:0006779	porphyrin-containing compound biosynthetic process	7	15.1	5.2E-04
GO:0030218	erythrocyte differentiation	12	6.5	5.5E-04
GO:0033014	tetrapyrrole biosynthetic process	7	13.4	7.3E-04
GO:0046501	protoporphyrinogen IX metabolic process	5	25.9	7.3E-04
GO:0015669	gas transport	6	17.3	7.3E-04
GO:0034101	erythrocyte homeostasis	12	6.0	7.3E-04
GO:0032446	protein modification by small protein conjugation	39	2.3	7.9E-04
CELLULAR COMPONENT^*^				
GO:0005833	hemoglobin complex	6	38.3	3.5E-06
GO:0030863	cortical cytoskeleton	11	9.2	1.0E-05
GO:0014731	spectrin-associated cytoskeleton	5	43.9	1.0E-05
GO:0005768	endosome	31	2.7	9.1E-05
GO:0005773	vacuole	26	2.9	2.4E-04
GO:0044448	cell cortex part	11	6.3	2.4E-04
GO:0005856	cytoskeleton	54	1.9	3.4E-04
GO:0036019	endolysosome	5	20.6	3.9E-04
GO:0031410	cytoplasmic vesicle	48	1.9	5.1E-04
GO:0097708	intracellular vesicle	48	1.9	5.1E-04
***MOLECULAR FUNCTION***				
GO:0046983	protein dimerization activity	41	2.10	9.4E-03
***KEGG PATHWAY***				n.s.

Top 10 enriched terms

n.s., not significant after Benjamini-Hochberg correction

Finally, the presence of a chronic *P*. *aeruginosa* infection negatively correlated with the eigengene of the magenta module. GO analysis revealed terms related to heme metabolic process and hemoglobin complex (**Table 5**). The magenta network comprised three hub genes encoding the following proteins: SLC25A39 (Solute Carrier Family 25 member 39), a mitochondrial solute carrier protein, GATA1 (GATA Binding Protein 1), an erythroid transcription factor and CDC34 (Cell Division Cycle 34), a ubiquitin-conjugating enzyme (**S2 Fig**).

## Discussion

CF patients present a clinical heterogeneity that is not fully explained by the type of mutations in the *CFTR* gene. Previous studies emphasized that other genes modulate the clinical phenotype and account for the development of comorbidities [1]. CF modifier genes were extensively searched in genetic studies first and more recently in transcriptomic studies [1].

Transcriptomic analyses in airway cell lines and nasal epithelial cell samples showed expression changes in genes involved in cell proliferation, inflammation and immune responses, protein metabolism, and calcium and membrane pathways [22–24]. More recently, blood samples from CF patients presenting either severe or mild lung disease were analyzed: genes of the type I interferon response and ribosomal stalk proteins were differentially expressed [25]. However, no healthy subjects were compared with CF patients in that study.

Herein, we used RNA-seq to profile blood samples from CF patients and healthy controls. An added value of this cohort is that the clinical data were recorded on the same day biological samples were collected. Importantly, in addition to the differential gene expression analysis, we implemented WGCNA.

DE genes between CF patients and controls were overrepresented among genes important for the leukocyte activation and the response to bacterial infection, which is consistent with the permanent inflammation and chronic infections in CF patients. KEGG analysis highlighted the Th17 lymphocytes activation pathway.

A limitation of the DE gene analysis is that a number of relevant genes do not reach significance after correction for multiple tests, unless large cohorts are assembled. But it is difficult to fulfill this condition in rare disease studies. To overcome this drawback, we set up WGCNA [15]. WGCNA detects networks of co-regulated and highly connected genes that belong to biological pathways and reduces the number of variables to be tested, thus decreasing the false discovery rate [15]. Using WGCNA, we found that clinical traits of interest in cystic fibrosis correlated with modules of co-expressed genes in blood samples.

Because lung disease is the main cause of mortality and morbidity in CF, first we inspected the dark turquoise module that correlated with the lung function (FEV_1_ and FVC) and BMI. Lung function and BMI are positively correlated in CF and their deterioration is predictive of patient decline and, ultimately, patient death [26]. The GO analysis of the dark turquoise module showed terms related to cell-cell adhesion and cell adherence junctions. Interestingly, the same association between lung function and cell adhesion was highlighted by the DNA methylation analysis of CF nasal epithelial cell samples [10].

In the present transcriptomic study, *FLNA* is a hub gene of the dark turquoise module. The Filamin A protein is an actin-binding and scaffolding protein that binds to integrins and also interacts with CFTR [27]. Of interest, mutations in the *FLNA* gene result in interstitial lung disease, a severe respiratory illness [28]. The FLNA protein is required for optimal T cell homing into lymph nodes and inflamed tissues [29]. To explain the association between lung function and cell adhesion in cystic fibrosis, we argue that if cell junctions are loosened, the leukocyte trafficking from the blood stream to the airways is facilitated, and the resulting high inflammation reduces lung function.

The decline of lung function is steeper in CF patients with diabetes and the fast decay starts 1–3 years before the appearance of diabetes [3]. Peaks of hyperglycemia also occur before the appearance of diabetes [3]. To explain the association between diabetes and a more rapid lung function decline, modules that correlated with lung function (dark turquoise) and with diabetes and HbA1c levels (light yellow) should be investigated together. Of interest, some of their respective hub genes encode proteins (FLNA and integrins) that bind one to each other to regulate T lymphocyte and neutrophil trafficking [26,30]. Also, high glucose modifies the levels of the Flna protein in rat endothelial cells [31]. All together, these findings suggest that glucose fluctuations can be the initial event that alter the expression of genes responsible for leukocyte trafficking, increases airway inflammation and, thereby, reduces the lung function in cystic fibrosis.

CF patients with diabetes may present microvascular complications, namely retinopathy and nephropathy [3]. Platelets are activated by glucose and their abnormalities are the initial event responsible for microvascular complications in diabetes [32]. Genes encoding platelet aggregation proteins were overrepresented in the light yellow module which, therefore, should be analyzed in detail with respect to these comorbidities.

The presence of diabetes but not HbA1c levels correlated with the cyan module. It comprised genes that encode proteins of the 20S and 19S proteasome subunits. A pivotal function of the proteasome is to degrade the oxidized and misfolded proteins that are generated by oxidative stress [33]. Through visualization of the most connected genes of this module, we identified *SNX17*, previously associated with glucose-homeostasis in muscle and adipose tissues of type 2 diabetic patients [34]. The SNX17 protein activates T cells by regulating T cell receptors and integrin recycling in humans [35]. *SNX17* is a hub connected to 12 co-expressed genes, namely *PARK7/DJ-1* encoding a protein deglycase and *ATP5B* encoding the mitochondrial ATP synthase B subunit. In diabetic mice, the expression of the ATP5B protein is high and activated by reactive oxygen species (ROS) [36]. Overall, genes of the cyan module encode proteins that are activated by and protect from high levels of ROS.

Adult CF patients are sensitized to chronic opportunistic airway infections. In the blood transcriptome dataset, *P*. *aeruginosa* chronic infection negatively correlated with the magenta module. This correlation should be taken with caution because only two patients were not chronically infected by *P*. *aeruginosa*. Genes of the magenta module encode proteins important for erythrocyte differentiation and homeostasis, and for the hemoglobin complex. *SLC25A39*, a hub in this module, codes for a mitochondrial solute carrier protein. Silencing of the mouse Slc25a39 ortholog affected iron incorporation, essential for bacterial growth [37,38]. Thus, the magenta module points to the role of iron fixation in *P*. *aeruginosa* infections.

The present study has some limitations. We analyzed gene transcription in blood samples from 33 CF patients and 16 healthy controls. Confirmatory studies should be carried out in independent cohorts. We showed that blood is an informative surrogate tissue to address the contribution of inflammation to CFRD. However, evidence exists that this comorbidity also depends on an intrinsic pancreatic islet dysfunction whose study requires access to pancreas samples. Finally, in the future, patients should be followed longitudinally to correlate the gene signatures with the progression of the disease.

## Conclusions

In summary, using blood samples from CF patients, we identified modules of co-expressed genes that belong to relevant biological pathways. Detailed inspection of three modules that correlated with the presence of diabetes and lung function pointed to cell adhesion, leukocyte trafficking and production of ROS as central mechanisms in CFRD and pulmonary function decline. A fourth module that correlated with *P*. *aeruginosa* infection comprised genes important for iron fixation. Of note, we showed that blood is an informative surrogate tissue to address the contribution of inflammation to lung disease and diabetes in CF patients. Finally, we provided evidence that WGCNA is much valuable to analyze–omic datasets in rare genetic diseases as patient cohorts are inevitably small.

## Supporting information

S1 Fig Biotype distribution and expression level of the corresponding transcripts. (TIFF)

S2 Fig Network representation of the magenta module. Top genes and their connections were visualized with Cytoscape [16]. Genes of interest were emphasized. The correlated clinical trait and main gene ontology (GO) term are shown.
(TIFF)

S1 Table Primer sequences and conditions used for qPCR validation. (DOCX)

S2 Table Differentially expressed genes between CF patients and controls. (DOCX)

S3 Table GO terms for differentially expressed genes between CF patients and controls. (DOCX)

S4 Table Genes and modules. Rows correspond to genes. A total of 15077 genes with FPKM > 0.1 in blood samples are listed. Columns list the gene name followed by module membership (kMEi) and corresponding p-values for each module of co-expressed genes. Lists of genes with high module membership can be sorted by selecting decreasing kMEi or increasing p-values. The kMEi is the correlation between the expression of a gene and the module eigengene. It ranges between 0 and 1. A gene is highly connected to other genes of a module when its kMEi approaches 1.
(XLSX)

## References

[1] Cutting GR. Cystic fibrosis genetics: from molecular understanding to clinical application. Nat Rev Genet. 2015;16(1):45–56. 10.1038/nrg3849 Review. . 25404111 PMC4364438

[2] Elborn JS. Cystic fibrosis. Lancet. 2016;388(10059):2519–2531. 10.1016/S0140-6736(16)00576-6 Review. . 27140670

[3] Bridges N, Rowe R, Holt RI. Unique challenges of cystic fibrosis-related diabetes. Diabet Med. 2018; 10.1111/dme.13652 Review. 29687501

[4] Delaspre F, Beer RL, Rovira M, Huang W, Wang G, Gee S, et al Centroacinar Cells Are Progenitors That Contribute to Endocrine Pancreas Regeneration. Diabetes. 2015;64:3499–34509. 10.2337/db15-0153 26153247 PMC4587647

[5] Edlund A, Esguerra JL, Wendt A, Flodström-Tullberg M, Eliasson L. CFTR and Anoctamin 1 (ANO1) contribute to cAMP amplified exocytosis and insulin secretion in human and murine pancreatic beta-cells. BMC Med. 2014;12:87 10.1186/1741-7015-12-87 24885604 PMC4035698

[6] Hart NJ, Aramandla R, Poffenberger G, Fayolle C, Thames AH, Bautista A, et al Cystic fibrosis-related diabetes is caused by islet loss and inflammation. JCI Insight. 2018;3:8, pii: 98240 10.1172/jci.insight.98240 29669939 PMC5931120

[7] Malhotra S, Hayes D Jr, Wozniak DJ. Cystic Fibrosis and Pseudomonas aeruginosa: the Host-Microbe Interface. Clin Microbiol Rev. 2019;32:3 pii: e00138–18. 10.1128/CMR.00138-18 Review. 31142499 PMC6589863

[8] Snouwaert JN, Brigman KK, Latour AM, Malouf NN, Boucher RC, Smithies O, et al An animal model for cystic fibrosis made by gene targeting. Science. 1992;257:1083–1088. 10.1126/science.257.5073.1083 1380723

[9] Magalhães M, Rivals I, Claustres M, Varilh J, Thomasset M, Bergougnoux A, et al DNA methylation at modifier genes of lung disease severity is altered in cystic fibrosis. Clin Epigenetics. 2017;9:19 10.1186/s13148-016-0300-8 eCollection 2017. 28289476 PMC5310067

[10] Magalhães M, Tost J, Pineau F, Rivals I, Busato F, Alary N, et al Dynamic changes of DNA methylation and lung disease in cystic fibrosis: lessons from a monogenic disease. Epigenomics. 2018; 10.2217/epi-2018-0005 30052057

[11] Kim D, Pertea G, Trapnell C, Pimentel H, Kelley R, Salzberg SL. TopHat2: accurate alignment of transcriptomes in the presence of insertions, deletions and gene fusions. Genome Biol. 2013;14(4):R36 10.1186/gb-2013-14-4-r36 23618408 PMC4053844

[12] Anders S and Huber W. Differential expression analysis for sequence count data. Genome Biol. 2010;11(10):R106 10.1186/gb-2010-11-10-r106 20979621 PMC3218662

[13] Anders S, Pyl PT and Huber W. HTSeq—a Python framework to work with high-throughput sequencing data. Bioinformatics. 2015;31(2):166–9. 10.1093/bioinformatics/btu638 Epub 2014 Sep 25. 25260700 PMC4287950

[14] Pfaffl MW. A new mathematical model for relative quantification in real-time RT-PCR. Nucleic Acids Res. 2001;29(9):e45 10.1093/nar/29.9.e45 11328886 PMC55695

[15] Langfelder P and Horvath S. WGCNA: an R package for weighted correlation network analysis. BMC Bioinformatics. 2008;9:559 10.1186/1471-2105-9-559 19114008 PMC2631488

[16] Shannon P, Markiel A, Ozier O, Baliga NS, Wang JT, Ramage D, et al Cytoscape: a software environment for integrated models of biomolecular interaction networks. Genome Research. 2003;13:2498–2504. 10.1101/gr.1239303 14597658 PMC403769

[17] Wang J, Duncan D, Shi Z, Zhang B. WEB-based GEne SeT AnaLysis Toolkit (WebGestalt): update 2013. Nucleic Acids Res. 2013;41:77–83. 10.1093/nar/gkt439 23703215 PMC3692109

[18] Debey-Pascher S1, Hofmann A, Kreusch F, Schuler G, Schuler-Thurner B, Schultze JL, et al RNA-stabilized whole blood samples but not peripheral blood mononuclear cells can be stored for prolonged time periods prior to transcriptome analysis. J Mol Diagn. 2011;13(4):452–460. 10.1016/j.jmoldx.2011.03.006 21704280 PMC3123794

[19] Quanjer PH, Stanojevic S, Cole TJ, Baur X, Hall GL, Culver BH, et al Multi-ethnic reference values for spirometry for the 3-95-yr age range: the global lung function 2012 equations. Eur Respir J. 2012;40(6):1324–1343. 10.1183/09031936.00080312 . 22743675 PMC3786581

[20] Muhlebach MS, Clancy JP, Heltshe SL, Ziady A, Kelley T, Accurso F, et al Biomarkers for cystic fibrosis drug development. J Cyst Fibros. 2016;15(6):714–723. 10.1016/j.jcf.2016.10.009 Review. 28215711 PMC5321565

[21] Calderwood DA, Huttenlocher A, Kiosses WB, Rose DM, Woodside DG, Schwartz MA, et al Increased filamin binding to beta-integrin cytoplasmic domains inhibits cell migration. Nat Cell Biol. 2001;3:1060–1068. 10.1038/ncb1201-1060 11781567

[22] Ogilvie V, Passmore M, Hyndman L, Jones L, Stevenson B, Wilson A, et al Differential global gene expression in cystic fibrosis nasal and bronchial epithelium. Genomics. 2011;98:327–336. 10.1016/j.ygeno.2011.06.008 21756994

[23] Wright JM, Merlo CA, Reynolds JB, Zeitlin PL, Garcia JG, Guggino WB, et al Respiratory epithelial gene expression in patients with mild and severe cystic fibrosis lung disease. Am J Respir Cell Mol Biol. 2006;35:327–336. 10.1165/rcmb.2005-0359OC 16614352 PMC2643286

[24] Clarke LA, Sousa L, Barreto C, Amaral MD. Changes in transcriptome of native nasal epithelium expressing F508del-CFTR and intersecting data from comparable studies. Respir Res. 2013;14:13 10.1186/1465-9921-14-13 23537407 PMC3637641

[25] Kormann MSD, Dewerth A, Eichner F, Baskaran P, Hector A, Regamey N, et al Transcriptomic profile of cystic fibrosis patients identifies type I interferon response and ribosomal stalk proteins as potential modifiers of disease severity. PLoS One. 2017;12(8):e0183526 10.1371/journal.pone.0183526 eCollection 2017. 28846703 PMC5573219

[26] Stephenson AL, Mannik LA, Walsh S, Brotherwood M, Robert R, Darling PB, et al Longitudinal trends in nutritional status and the relation between lung function and BMI in cystic fibrosis: a population-based cohort study. Am J Clin Nutr. 2013;97:872–877. 10.3945/ajcn.112.051409 23388659

[27] Playford MP1, Nurminen E, Pentikäinen OT, Milgram SL, Hartwig JH, Stossel TP, et al Cystic fibrosis transmembrane conductance regulator interacts with multiple immunoglobulin domains of filamin A. J Biol Chem. 2010;285:17156–17165. 10.1074/jbc.M109.080523 20351098 PMC2878090

[28] Shelmerdine SC, Semple T, Wallis C, Aurora P, Moledina S, Ashworth MT, et al Filamin A (FLNA) mutation-A newcomer to the childhood interstitial lung disease (ChILD) classification. Pediatr Pulmonol. 2017;52:1306–1315. 10.1002/ppul.23695 28898549

[29] Savinko T, Guenther C, Uotila LM, Llort Asens M, Yao S, Tojkander S, et al Filamin A Is Required for Optimal T Cell Integrin-Mediated Force Transmission, Flow Adhesion, and T Cell Trafficking. J Immunol. 2018;200:3109–3116. 10.4049/jimmunol.1700913 29581355

[30] Uotila LM, Guenther C, Savinko T, Lehti TA, Fagerholm SC. Filamin A Regulates Neutrophil Adhesion, Production of Reactive Oxygen Species, and Neutrophil Extracellular Trap Release. J Immunol. 2017;199:3644–3653. 10.4049/jimmunol.1700087 28986439

[31] Lee HZ, Yeh FT, Wu CH. The effect of elevated extracellular glucose on adherens junction proteins in cultured rat heart endothelial cells. Life Sci. 2004;74:2085–2096. 10.1016/j.lfs.2003.06.046 14969714

[32] Di Mario U, Borsey DQ, Contreas G, Prowse CV, Clarke BF, Andreani D. The relationship of soluble immune complexes, insulin antibodies and insulin-anti-insulin complexes to platelet and coagulation factors in type 1 diabetic patients with and without proliferative retinopathy. Clin Exp Immunol. 1986;65:57–65. 2947762 PMC1542274

[33] Tanaka K. The proteasome: overview of structure and functions. Proc Jpn Acad Ser B Phys Biol Sci. 2009;85:12–36. Review. 10.2183/pjab.85.12 19145068 PMC3524306

[34] Sajuthi SP, Sharma NK, Chou JW, Palmer ND, McWilliams DR, Beal J, et al Mapping adipose and muscle tissue expression quantitative trait loci in African Americans to identify genes for type 2 diabetes and obesity. Hum Genet. 2016;135(8):869–80. 10.1007/s00439-016-1680-8 27193597 PMC4947558

[35] Osborne DG, Piotrowski JT, Dick CJ, Zhang JS, Billadeau DD. SNX17 affects T cell activation by regulating TCR and integrin recycling. J Immunol. 2015;194:4555–4566. 10.4049/jimmunol.1402734 25825439 PMC4402276

[36] Guan SS, Sheu ML, Wu CT, Chiang CK, Liu SH. ATP synthase subunit-β down-regulation aggravates diabetic nephropathy. Sci Rep. 2015;5:14561 10.1038/srep14561 26449648 PMC4598833

[37] Shaw GC, Cope JJ, Li L, Corson K, Hersey C, Ackermann GE, et al Mitoferrin is essential for erythroid iron assimilation. Nature. 2006;440:96–100. 10.1038/nature04512 16511496

[38] Minandri F, Imperi F, Frangipani E, Bonchi C, Visaggio D, Facchini M, et al Role of Iron Uptake Systems in Pseudomonas aeruginosa Virulence and Airway Infection. Infect Immun. 2016;84:2324–2335. 10.1128/IAI.00098-16 27271740 PMC4962624

