# Supplementary figures and images for: Blood co-expression modules identify potential modifier genes of diabetes and lung function in cystic fibrosis
Source: PLoS One. 2020 Apr 17;15(4):e0231285. doi: 10.1371/journal.pone.0231285 (PMC7164665; doi:10.1371/journal.pone.0231285)

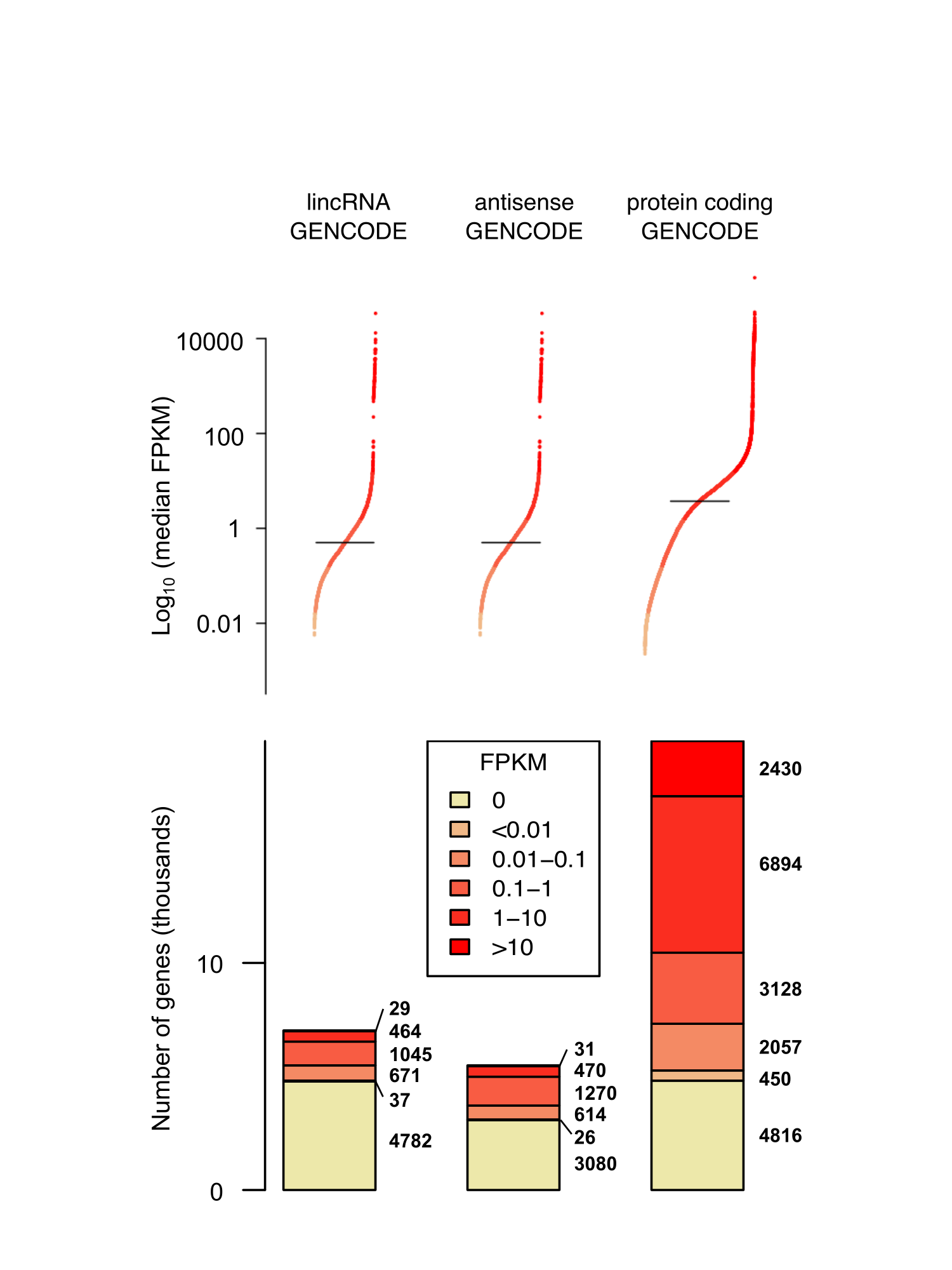

Supplement: S1 Fig — (TIFF) [file pone.0231285.s001.tiff]

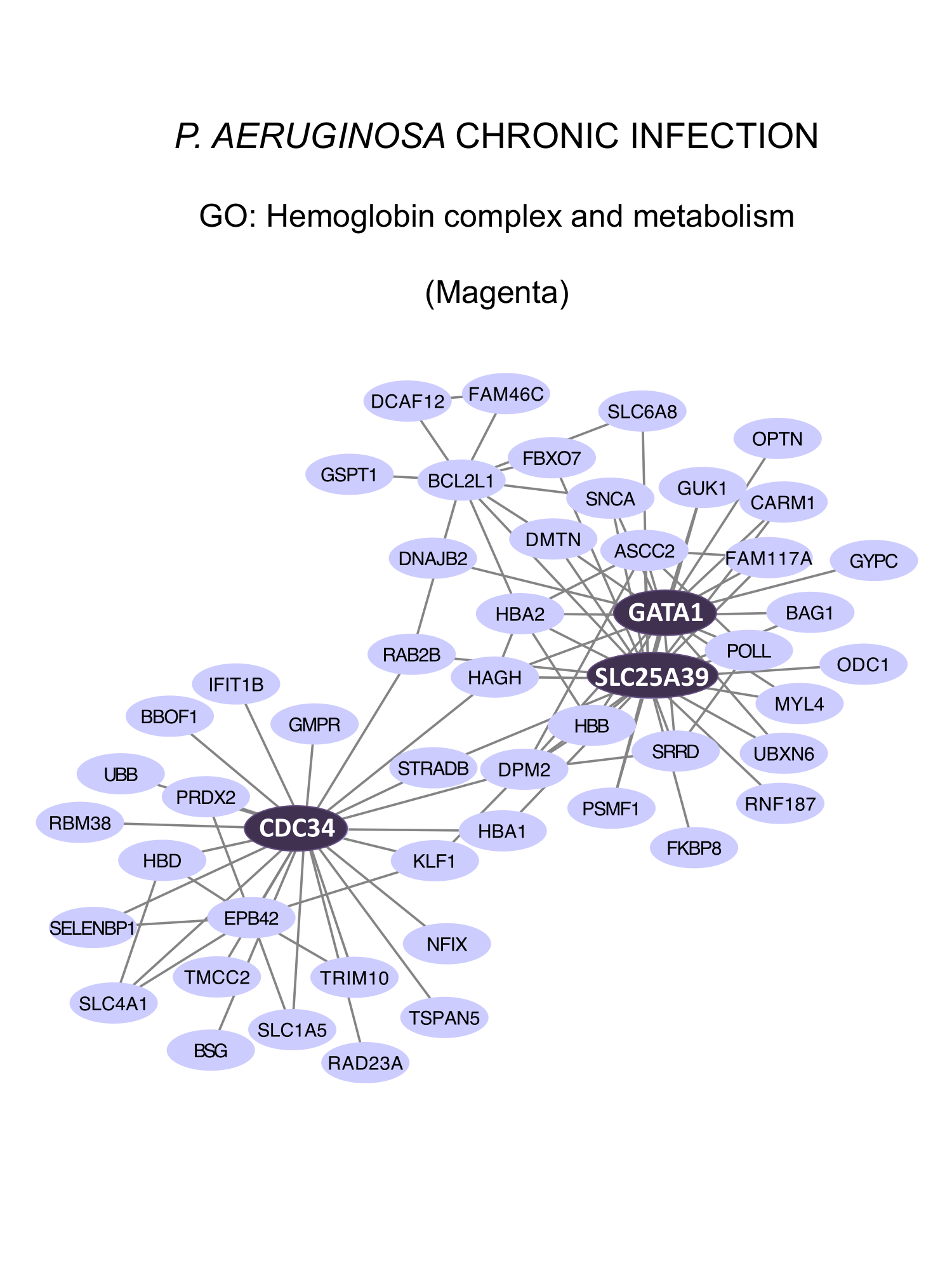

Supplement: S2 Fig — Top genes and their connections were visualized with Cytoscape [16]. Genes of interest were emphasized. The correlated clinical trait and main gene ontology (GO) term are shown. (TIFF) [file pone.0231285.s002.tiff]
