# Supplementary material for: Blood co-expression modules identify potential modifier genes of diabetes and lung function in cystic fibrosis
Source: PLoS One. 2020 Apr 17;15(4):e0231285. doi: 10.1371/journal.pone.0231285 (PMC7164665; doi:10.1371/journal.pone.0231285)
Supplement: S1 Table — (DOCX) [file pone.0231285.s003.docx]

**S1 Table.** Primer sequences and conditions used for qPCR validation

| **Gene** | **Biotype** | **Gene position** | **Primer sequences (5'-3')** | **Design position** | **Amplicon size** | **Annealing temperature** | **Primers concentration** |
| --- | --- | --- | --- | --- | --- | --- | --- |
| ALPL | Protein coding | chr1:21835851-21904905 | F: GACCTCCTCGGAAGACACTC | Exon 12 / exon 13 | 180 bp | 60°C | 0.25 µM |
| NM_000478 |  |  | R: CCCACCACCTTGTAGCCAG |  |  |  |  |
| CITF22-49E9.3 | LincRNA | chr22:50326846-50327722 | F: GCCCCTGTCTGCGGTGG | Exon 1 | 94 bp | 60°C | 0.25µM |
|  |  |  | R: CCTCTGCTGCTCTGTTTCACG |  |  |  |  |
| CLEC4D | Protein coding | chr12:8,666,136-8,674,960 | F: GACTCATCACAACTTTTCACGC | Exon 3 / exon 4 | 131 bp | 60°C | 0.25 µM |
| NM_080387 |  |  | R: GACAACAGTTCCAGGTGCTC |  |  |  |  |
| TLR5 | Protein coding | chr1:223,282,748-223,316,624 | F: CCTCTGCCCCTAGAATAAGAAC | Exon 5 / exon 6 | 241 bp | 60°C | 0.25 µM |
| NM_003268 |  |  | R: GACTGTCCTGATATAGTTGAAGC |  |  |  |  |
| YWHAZ | Protein coding | chr8:101,930,804-101,962,799 | F: AAAAGACGGAAGGTGCTGAG | Exon 2 / exon 3 | 149 bp | 60°C | 0.5 µM |
| NM_001135702 |  |  | R: GACTTTGCTCTCTGCTTGTG |  |  |  |  |
