# Supplementary material for: Blood co-expression modules identify potential modifier genes of diabetes and lung function in cystic fibrosis
Source: PLoS One. 2020 Apr 17;15(4):e0231285. doi: 10.1371/journal.pone.0231285 (PMC7164665; doi:10.1371/journal.pone.0231285)
Supplement: S2 Table — (DOCX) [file pone.0231285.s004.docx]

**S2 Table**. Differentially expressed genes between CF patients and controls

| **Gene** | **Gene biotype** | **C median FPKM** | **CF median FPKM** | **log_2_FC CF/C^*^** | **FDR** |
| --- | --- | --- | --- | --- | --- |
| CHGB | protein_coding | 0.012 | 0.562 | 3.47 | 4.60E-02 |
| CHGA | protein_coding | 0.005 | 0.499 | 3.30 | 4.10E-02 |
| DLK1 | protein_coding | 0.002 | 0.109 | 2.86 | 1.90E-02 |
| NR2E1 | protein_coding | 0.005 | 0.149 | 2.46 | 3.40E-02 |
| CLRN1-AS1 | antisense | 0.024 | 0.172 | 2.29 | 1.50E-02 |
| DBH | protein_coding | 0.003 | 0.127 | 2.28 | 3.80E-02 |
| CHRNA3 | protein_coding | 0.001 | 0.024 | 1.80 | 2.70E-02 |
| GALNT14 | protein_coding | 0.679 | 3.103 | 1.77 | 2.70E-02 |
| CBS | protein_coding | 0.015 | 0.061 | 1.75 | 1.30E-02 |
| SLC26A8 | protein_coding | 0.74 | 2.733 | 1.72 | 1.30E-02 |
| TH | protein_coding | 0 | 0.032 | 1.72 | 3.30E-02 |
| UNQ6494 | lincRNA | 0.061 | 0.254 | 1.71 | 1.80E-02 |
| RP11-166N6.2 | antisense | 0.773 | 3.415 | 1.65 | 1.90E-02 |
| CTD-3203P2.3 | antisense | 0.13 | 0.407 | 1.62 | 4.70E-02 |
| S100A12 | protein_coding | 54.631 | 230.453 | 1.61 | 4.10E-03 |
| RP11-10A14.5 | lincRNA | 0.002 | 0.1 | 1.61 | 4.40E-02 |
| SYN2 | protein_coding | 0.019 | 0.081 | 1.60 | 3.40E-02 |
| PCSK1 | protein_coding | 0.005 | 0.023 | 1.58 | 3.90E-02 |
| RP11-531H8.2 | lincRNA | 4.982 | 14.923 | 1.57 | 2.50E-02 |
| UCHL1 | protein_coding | 0.012 | 0.059 | 1.55 | 1.80E-02 |
| ANXA3 | protein_coding | 4.798 | 16.804 | 1.48 | 2.30E-02 |
| SOCS3 | protein_coding | 12.617 | 43.924 | 1.45 | 3.30E-02 |
| RP11-531H8.1 | lincRNA | 0.021 | 0.151 | 1.43 | 3.80E-02 |
| HM13-AS1 | antisense | 0.13 | 0.358 | 1.41 | 3.80E-02 |
| CAPN13 | protein_coding | 0.053 | 0.139 | 1.39 | 4.40E-02 |
| KREMEN1 | protein_coding | 3.597 | 9.95 | 1.38 | 4.60E-02 |
| RP11-231D20.2 | antisense | 0.009 | 0.036 | 1.35 | 2.30E-02 |
| SH3RF3-AS1 | lincRNA | 0.051 | 0.133 | 1.34 | 4.80E-02 |
| FSTL4 | protein_coding | 0.03 | 0.07 | 1.33 | 1.50E-02 |
| KAZN | protein_coding | 0.216 | 0.634 | 1.30 | 2.30E-02 |
| RP11-20B24.4 | antisense | 0.14 | 0.42 | 1.29 | 4.60E-02 |
| HBM | protein_coding | 7.928 | 21.623 | 1.28 | 4.40E-02 |
| GS1-590J6.3 | lincRNA | 0.138 | 0.376 | 1.28 | 5.00E-02 |
| PGLYRP1 | protein_coding | 15.947 | 48.328 | 1.22 | 2.50E-02 |
| RP11-239E10.2 | lincRNA | 0.04 | 0.121 | 1.22 | 3.80E-02 |
| CLEC4D | protein_coding | 8.483 | 26.352 | 1.19 | 2.30E-02 |
| TLR5 | protein_coding | 5.789 | 15.041 | 1.17 | 1.70E-02 |
| RP11-556I13.2 | antisense | 2.475 | 6.722 | 1.16 | 4.00E-02 |
| CITF22-49E9.3 | lincRNA | 5.002 | 10.471 | 1.14 | 1.30E-02 |
| ST3GAL4-AS1 | antisense | 2.024 | 5.648 | 1.14 | 4.10E-02 |
| S100A8 | protein_coding | 242.457 | 619.977 | 1.13 | 1.80E-02 |
| SLC37A3 | protein_coding | 2.437 | 7.241 | 1.13 | 2.40E-02 |
| F5 | protein_coding | 5.559 | 14.553 | 1.11 | 1.90E-02 |
| FCAR | protein_coding | 10.05 | 26.384 | 1.10 | 2.00E-02 |
| ALPL | protein_coding | 21.736 | 56.294 | 1.10 | 4.20E-02 |
| NAIP | protein_coding | 1.673 | 4.272 | 1.08 | 2.10E-02 |
| ADM | protein_coding | 5.99 | 13.538 | 1.08 | 3.30E-02 |
| CTB-61M7.2 | lincRNA | 7.69 | 19.937 | 1.06 | 4.00E-02 |
| DSC2 | protein_coding | 3.082 | 6.382 | 1.05 | 1.20E-02 |
| SDR16C5 | protein_coding | 0.072 | 0.026 | -1.59 | 4.70E-02 |
| RP11-731F5.2 | lincRNA | 0.513 | 0.23 | -1.55 | 2.50E-02 |
| IL23R | protein_coding | 0.717 | 0.29 | -1.54 | 1.20E-02 |
| SLC4A10 | protein_coding | 3.159 | 1.145 | -1.52 | 4.30E-03 |
| LINGO2 | protein_coding | 0.353 | 0.149 | -1.50 | 1.30E-02 |
| BCAS1 | protein_coding | 0.072 | 0.032 | -1.45 | 7.40E-03 |
| BOK | protein_coding | 0.507 | 0.202 | -1.40 | 2.80E-02 |
| ENHO | protein_coding | 0.685 | 0.308 | -1.39 | 1.20E-02 |
| TEAD2 | protein_coding | 0.11 | 0.038 | -1.36 | 1.30E-02 |
| NTN4 | protein_coding | 0.228 | 0.114 | -1.34 | 3.40E-02 |
| LGALS9B | protein_coding | 0.204 | 0.085 | -1.32 | 1.20E-02 |
| LTK | protein_coding | 1.042 | 0.456 | -1.24 | 4.10E-03 |
| ADAM12 | protein_coding | 0.305 | 0.142 | -1.23 | 1.10E-02 |
| COL13A1 | protein_coding | 0.139 | 0.064 | -1.22 | 3.40E-02 |
| ANKUB1 | protein_coding | 0.561 | 0.27 | -1.21 | 4.10E-03 |
| RORC | protein_coding | 1.32 | 0.635 | -1.14 | 4.10E-03 |
| RASSF6 | protein_coding | 0.219 | 0.102 | -1.14 | 1.20E-02 |
| TNFRSF13B | protein_coding | 0.338 | 0.15 | -1.13 | 4.10E-03 |
| GPM6A | protein_coding | 0.238 | 0.11 | -1.11 | 1.70E-02 |
| PAK6 | protein_coding | 0.082 | 0.039 | -1.11 | 3.90E-02 |
| LINGO4 | protein_coding | 0.068 | 0.035 | -1.10 | 4.60E-02 |
| COL4A3 | protein_coding | 0.444 | 0.199 | -1.09 | 1.20E-02 |
| ADAMTS1 | protein_coding | 0.731 | 0.405 | -1.06 | 1.80E-02 |
| COL4A4 | protein_coding | 0.35 | 0.164 | -1.03 | 4.10E-03 |
| CTD-2509G16.5 | lincRNA | 0.538 | 0.267 | -1.02 | 2.40E-02 |
| PRICKLE2 | protein_coding | 0.078 | 0.037 | -1.02 | 4.40E-02 |
| MIR181A2HG | antisense | 1.603 | 0.919 | -1.01 | 1.90E-02 |

^*^ log_2_FoldChange ≤ -1 or ≥ 1 is equivalent to a minimum 2-fold change (in either direction).
