# Supplementary material for: Blood co-expression modules identify potential modifier genes of diabetes and lung function in cystic fibrosis
Source: PLoS One. 2020 Apr 17;15(4):e0231285. doi: 10.1371/journal.pone.0231285 (PMC7164665; doi:10.1371/journal.pone.0231285)
Supplement: S3 Table — (DOCX) [file pone.0231285.s005.docx]

| **S3 Table.** GO terms for differentially expressed genes between CF patients and controls | | | | | | | | |
| --- | --- | --- | --- | --- | --- | --- | --- | --- |
|  | |  | | |  | | |  |
| **Geneset** | **Description** | **Number of genes** | **Ratio of enrichment** | **FDR (B-H)** | | |  |  |
| ***BIOLOGICAL PROCESS*†** | |  | | |  |  |  |  |
| GO:0009617 | response to bacterium | 19 | 5.5 | 1.2E-05 | | |  |  |
| GO:0009607 | response to biotic stimulus | 23 | 4.1 | 3.3E-05 | | |  |  |
| GO:0051707 | response to other organism | 22 | 4.1 | 3.4E-05 | | |  |  |
| GO:0043207 | response to external biotic stimulus | 22 | 4.1 | 3.4E-05 | | |  |  |
| GO:0006955 | immune response | 30 | 3.0 | 7.3E-05 | | |  |  |
| GO:0009605 | response to external stimulus | 35 | 2.5 | 1.8E-04 | | |  |  |
| GO:0002366 | leukocyte activation involved in immune response | 11 | 7.3 | 3.4E-04 | | |  |  |
| GO:0002263 | cell activation involved in immune response | 11 | 7.2 | 3.4E-04 | | |  |  |
| GO:0042742 | defense response to bacterium | 11 | 7.2 | 3.4E-04 | | |  |  |
| GO:0032496 | response to lipopolysaccharide | 12 | 5.7 | 1.1E-03 | | |  |  |
| ***CELLULAR COMPONENT*†** | |  | | |  |  |  |  |
| GO:0005604 | basement membrane | 6 | 12.8 | 7.8E-03 | | |  |  |
| GO:0044420 | extracellular matrix component | 6 | 9.7 | 1.8E-02 | | |  |  |
| GO:0044433 | cytoplasmic vesicle part | 15 | 3.1 | 2.8E-02 | | |  |  |
| GO:0099503 | secretory vesicle | 10 | 4.2 | 2.8E-02 | | |  |  |
| GO:0005605 | basal lamina | 3 | 27.3 | 2.8E-02 | | |  |  |
| GO:0031410 | cytoplasmic vesicle | 21 | 2.3 | 2.8E-02 | | |  |  |
| GO:0097708 | intracellular vesicle | 21 | 2.3 | 2.8E-02 | | |  |  |
| GO:0005887 | integral component of plasma membrane | 20 | 2.3 | 3.1E-02 | | |  |  |
| GO:0043025 | neuronal cell body | 9 | 4.2 | 3.4E-02 | | |  |  |
| GO:0031226 | intrinsic component of plasma membrane | 20 | 2.3 | 3.7E-02 | | |  |  |
| ***MOLECULAR FUNCTION*** | |  | | |  |  |  |  |
| ns |  |  |  |  | | |  |  |
| ***KEGG PATHWAY*** | |  | | |  |  |  |  |
| hsa05321 | Inflammatory bowel disease (IBD) - Homo sapiens (human) | 6 | 10.9 | 4.6E-03 | | |  |  |
| hsa04659 | Th17 cell differentiation - Homo sapiens (human) | 7 | 7.7 | 4.6E-03 | | |  |  |

† Top 10 terms.

ns: not significant after Benjamini-Hochberg (B-H) correction.
