# Supplementary material for: Blood co-expression modules identify potential modifier genes of diabetes and lung function in cystic fibrosis
Source: PLoS One. 2020 Apr 17;15(4):e0231285. doi: 10.1371/journal.pone.0231285 (PMC7164665; doi:10.1371/journal.pone.0231285)
Supplement: Attachment — Submitted filename: Response to Reviewers.docx [file pone.0231285.s007.docx]

Dr Barbara Bardoni

Academic Editor

PLOS ONE

March the 12^th^, 2020

Dear Dr Bardoni,

I thank you for giving us the opportunity to submit a revised version of “Blood co-expression modules identify potential modifier genes of diabetes and lung function in cystic fibrosis” (PONE-D-20-00190).

In response to the reviewer’s remarks, we have revised the manuscript and provided a point-by-point discussion.

We hope that these comments are helpful to you and the revised version of our manuscript is suitable to be published in *PLOS One.*

Sincerely,

Albertina De Sario (PhD)

**Response to the Editor**

When submitting your revision, we need you to address these additional requirements:

1. Please ensure that your manuscript meets PLOS ONE's style requirements, including those for file naming. The PLOS ONE style templates can be found at http://www.plosone.org/attachments/PLOSOne_formatting_sample_main_body.pdf and <http://www.plosone.org/attachments/PLOSOne_formatting_sample_title_authors_affiliations.pdf>

We followed Plos One guidelines.

2. We note that you have included the phrase “data not shown” in your manuscript. Unfortunately, this does not meet our data sharing requirements. PLOS does not permit references to inaccessible data. We require that authors provide all relevant data within the paper, Supporting Information files, or in an acceptable, public repository. Please add a citation to support this phrase or upload the data that corresponds with these findings to a stable repository (such as Figshare or Dryad) and provide and URLs, DOIs, or accession numbers that may be used to access these data. Or, if the data are not a core part of the research being presented in your study, we ask that you remove the phrase that refers to these data.

In the revised manuscript, data not shown were removed. Line 246, we quote the correlation coefficient and p-value between dark turquoise module and FVC. Line 292, we quoted the supplementary Figure 2 showing the top genes of the magenta module.

3. We note that you have stated that you will provide repository information for your data at acceptance. Should your manuscript be accepted for publication, we will hold it until you provide the relevant accession numbers or DOIs necessary to access your data. If you wish to make changes to your Data Availability statement, please describe these changes in your cover letter and we will update your Data Availability statement to reflect the information you provide.

Normalized data and raw data generated during the current study are available in Gene Expression Omnibus (GEO) with accession number 136371 (<https://www.ncbi.nlm.nih.gov/geo/query/acc.cgi?acc=GSE136371>). Data will be made available for all as soon as the manuscript is accepted for publication.


**Response to Reviewer #1:**

This study used transcriptome analysis of blood samples from individuals with CF in an attempt to find novel gene modifiers of CF disease. Using a Weighted Gene Correlation Network Analysis (WGCNA) approach, Lung function, BMI, diabetes presence and chronic P. aeruginosa infection were found to be correlated with modules of co-expressed genes. The study is clear and well presented. I have only a few minor comments which, if addressed, would render the article suitable for publication.

Comments:
1. Methods p.5-6 (Table 1): It would be informative to have the standard error (+/- SEM) for values in this table, rather than just the mean or median values, in order to better assess the similarity between the two patient sets.

In Table 1 of the revised manuscript, we added the interquartile ranges (IQR).

2. Methods p.7 line 131: primer efficiency was determined to be 93% - presumably this means at least 93%?

Yes, it does mean at least 93%. Line 123 we added “at least”.

3. Methods p. 7-8 (WGCNA section): this section is not very informative for readers who have not used this method of data analysis, and would benefit from a slight expansion to clarify some of the terms.

Lines 135-152, we have summed up the main steps of WGCNA and slightly expanded the paragraph.

4. Results p 9-10: When presenting results for GO term enrichment a few of the most enriched groups are mentioned here along with a few of the representative genes. It would be more informative if the p values and number of genes in each group were shown in this section to reduce the necessity for looking at the supplementary table.

Lines 193-197, we added the p-values and the number of genes for GO terms.

5. Results p 10 (Validation by real time PCR): the validation is well described and the results seem to be sound but the number of DE genes/lincRNAs validated is rather low, given the relative ease and rapidity of this technique. Particularly, given the non-validation of the lincRNA in the replication, I wonder why the authors did not try validating one of the other transcripts.

For real-time PCR validation, we selected 4 genes of interest having high, medium and low levels of expression. The lncRNA had rather low levels of expression in blood and was technically validated by real-time PCR in the discovery set of patients, the same used for RNAseq. In the confirmatory set of patients, although it did not reach significance, the lncRNA had the same direction of differential expression (overexpression in CF patients) and a similar fold-change as in RNAseq.

6. Results (WGCNA section including tables 3-4 and Figs 2-3): this section necessarily contracts the data in order to focus on a few modules of co-expressed genes that correlate with some CF parameters. However, it would be useful to provide a further clarification about the identity of other undiscussed modules which can be seen in Fig. 2. Table S4 is uninformative in this respect, as it is not easy to find the relevant genes in each module, even using the table legend on page 26. Either explain better how the data can be treated to find these genes, or provide another sheet in table S4 with the columns filtered to identify the important genes of each module.

In the revised manuscript, we have added the KEGG analysis of the 28 modules and provided evidence that gene modules are biologically meaningful. See lines 222-225 and Table 3 showing the KEGG analysis of 28 modules.

In the revised legend of Table S4, we have specified how to identify the most connected genes of a module and we have better explained the kMEi (lines 554-560). We wish to keep Table S4 as a whole, including modules that were not analyzed in details and genes that are below our thresholds. This information can be useful for other studies or people interested in other genes.

7. Discussion p 16-19. The discussion of the suitability of the blood transcriptome for studying the contribution of DE gene expression in CFRD should be extended. In particular it would be interesting to know what proportions of which cell types were present in the whole blood from which the RNA samples were extracted. Some cytological data from similar blood samples to those used in the study would help in this respect, and would allow a fuller appreciation of the functional significance of the data.

In the result section, we have provided blood cell composition for a subset of CF patients. Blood cell composition was within normal ranges, as expected for CF patients without exacerbation. In addition, we have specified that whole blood samples had been gathered in PAXgene tubes that not only stabilize RNA, but also preserve all types of circulating leukocytes. This is not the case for other blood storage methods that alter the cell proportion in blood samples and also the transcriptomic profile. A bibliographic reference has been provided. See lines 162-169 and reference 18.

We preferred to treat the issue in the result section to avoid expanding the discussion in line with referee 2 comments.

Reviewer #2: In this manuscript, the authors performed RNA-seq analysis of blood samples obtained from patients affected with cystic fibrosis and healthy controls. They analyzed the data obtained by Weighted Gene Correlation Network Analysis and they found a correlation between lung function, body mass index, the presence of diabetes, and chronic P. aeruginosa infections with four modules of co-expressed genes.

This study is original and overall well written, I found the discussion a little bit lengthy. Figure 3 is too dense.

We thank the reviewer for his comments. The discussion was slightly shortened and a few paragraphs were rephrased. Figure 3 was split into figure 3 and figure 4.

Figure legends are not included in the PDF file.

Figure legends are embedded in the manuscript according to PLOS One guidelines.

6. PLOS authors have the option to publish the peer review history of their article ([what does this mean?](https://journals.plos.org/plosone/s/editorial-and-peer-review-process#loc-peer-review-history)). If published, this will include your full peer review and any attached files.


If you choose “no”, your identity will remain anonymous but your review may still be made public.


**Do you want your identity to be public for this peer review?** For information about this choice, including consent withdrawal, please see our [Privacy Policy](https://www.plos.org/privacy-policy).

Reviewer #1: No

Reviewer #2: Yes: Enzo Lalli


[NOTE: If reviewer comments were submitted as an attachment file, they will be attached to this email and accessible via the submission site. Please log into your account, locate the manuscript record, and check for the action link "View Attachments". If this link does not appear, there are no attachment files to be viewed.]

While revising your submission, please upload your figure files to the Preflight Analysis and Conversion Engine (PACE) digital diagnostic tool, <https://pacev2.apexcovantage.com/>. PACE helps ensure that figures meet PLOS requirements. To use PACE, you must first register as a user. Registration is free. Then, login and navigate to the UPLOAD tab, where you will find detailed instructions on how to use the tool. If you encounter any issues or have any questions when using PACE, please email us at [figures@plos.org](mailto:figures@plos.org). Please note that Supporting Information files do not need this step.

Figures 1 to 4 have been uploaded to the PACE tool.
